# A supertree pipeline for summarizing phylogenetic and taxonomic information for millions of species

**DOI:** 10.7717/peerj.3058

**Published:** 2017-03-01

**Authors:** Benjamin D. Redelings, Mark T. Holder

**Affiliations:** 1 Department of Biology, Duke University, Durham, NC, United States; 2 Department of Ecology and Evolutionary Biology, University of Kansas, Lawrence, KS, United States; 3 Biodiversity Institute, University of Kansas, Lawrence, KS, United States; 4 Heidelberg Institute for Theoretical Studies, Heidelberg, Germany

**Keywords:** Supertree, Phylogenetics, Taxonomy, Software, Tree of life

## Abstract

We present a new supertree method that enables rapid estimation of a summary tree on the scale of millions of leaves. This supertree method summarizes a collection of input phylogenies and an input taxonomy. We introduce formal goals and criteria for such a supertree to satisfy in order to transparently and justifiably represent the input trees. In addition to producing a supertree, our method computes annotations that describe which grouping in the input trees support and conflict with each group in the supertree. We compare our supertree construction method to a previously published supertree construction method by assessing their performance on input trees used to construct the Open Tree of Life version 4, and find that our method increases the number of displayed input splits from 35,518 to 39,639 and decreases the number of conflicting input splits from 2,760 to 1,357. The new supertree method also improves on the previous supertree construction method in that it produces no unsupported branches and avoids unnecessary polytomies. This pipeline is currently used by the Open Tree of Life project to produce all of the versions of project’s “synthetic tree” starting at version 5. This software pipeline is called “*propinquity*”. It relies heavily on “*otcetera*”—a set of C++ tools to perform most of the steps of the pipeline. All of the components are free software and are available on GitHub.

## Background

The Open Tree of Life project seeks to build a platform for summarizing what is known about phylogenetic relationships across all of Life (Hinchliff et al., 2015). One primary goal of the project is to build a summary tree from a comprehensive taxonomic tree and a set of published trees. The summary tree is intended to transparently and justifiably represent phylogenetic information from these inputs. The taxonomic tree is derived from the Open Tree Taxonomy (OTT hereafter; publication in preparation). The phylogenetic inputs are published trees that have been curated to align the tips to OTT and to identify the correct rooting (see McTavish et al., 2015 for further details of the curation tools). Unlike OTT, these phylogenetic trees do not include all leaf taxa. The inputs (taxonomy and phylogenetic trees) and the output summary supertree are all rooted. Here we describe the software pipeline (*propinquity*) that summarizes and integrates these smaller source trees and the taxonomy tree into a single supertree and the noteworthy tools for manipulating and solving supertrees in the *otcetera* package.

### Goals

Translating the goals of the Open Tree of Life’s summary tree into an explicit set of criteria is not trivial. The summary supertree should represent the phylogenetic information from source trees in a transparent and justifiable fashion. We would like to allow users to correct errors in the supertree by improving the input information rather than requiring modification to the supertree algorithm. The pipeline was designed to create a tree which:

1. displays no unsupported groups,
2. defers to groupings from higher ranked trees in the case of conflict,
3. contains no unnecessary polytomies, and
4. displays as many groupings from input trees as possible.

These goals are described more fully below. In order to accomplish transparency and justification, our pipeline also produces annotations files with information about conflict and support.

#### Goal 1: each grouping is supported by at least one input

We require that each edge in the supertree be supported by at least one input tree edge. In addition to aiding interpretability, this requirement keeps the supertree from arbitrarily representing information that comes from none of the input trees. Of course, in a supertree analysis, the full tree will imply some relationships for subsets of the taxa that are not found in any input tree. So, the meaning of “supported by” needs some clarification.

**Notation, terminology, and the definition of “supported by”** Cutting any edge *j* of a rooted tree induces a bi-partition *S*(*j*) of the leaf taxa into two connected groups *S*_1_(*j*) and *S*_2_(*j*). Such a bi-partition is called a rooted split and written *S*(*j*) = *S*_1_(*j*)|•*S*_2_(*j*). Here *S*_1_(*j*) is called the “cluster” of *j*, and contains tip taxa on the side of edge *j* that does not contain the root. *S*_2_(*j*) contains the tip taxa on the side of edge *j* that contains the root. We refer to *S*(*j*) as a *rooted* split because the right side of the split implicitly contains the root node, as indicated by the •.

For any two rooted splits *A* = *A*_1_|•*A*_2_ and *B* = *B*_1_|•*B*_2_, we say that *A displays B* if *B*_1_⊆*A*_1_ and *B*_2_⊆*A*_2_. We also say that *A* and *B conflict* if none of *A*_1_∩*B*_1_, *A*_1_∩*B*_2_, and *B*_2_∩*A*_1_ are empty. Note that the intersection of the right sides of the splits is considered to be non-empty even if *A*_2_∩*B*_2_ is empty, because the right sides implicitly contain the root. If *A* and *B* do not conflict, then we say that they are *compatible*.^1^ 1It is possible to construct a rooted tree that displays both *A* and *B* if and only if *A* and *B* are compatible.If *A* displays *B*, then *A* and *B* must be compatible, since *A*_1_∩*B*_2_ and *B*_1_∩*A*_2_ must be empty. We adopt the shorthand of referring to an edge *k* when we mean the split *S*(*k*). So, when we say that edge *j* conflicts with edge *k*, we mean that *S*(*j*) conflicts with *S*(*k*).

Let 𝕊 denote a supertree, and *T*_*i*_ denote the *i*th input tree. We say that 𝕊 displays an edge *j* of *T*_*i*_ if any edge of 𝕊 displays *j*. We say that 𝕊 conflicts with a edge *j* of *T*_*i*_ if any edge of 𝕊 conflicts with *j*; otherwise 𝕊 is compatible with *j*. We say that edge *k* of 𝕊 is *supported by* edge *j* of *T*_*i*_ if 𝕊 displays *j*, but 𝕊 would not display *j* if we contracted edge *k*. We also adopt the shorthand of referring to edges by their tipward endpoint. By combining this with the above shorthand, we could say that a node *j* conflicts or does not conflict with another node *k*.

The set of taxa that are mapped to the tips of the tree *T*_*i*_ is $\mathcal{L}(i)$. 𝕊(*i*) denotes the summary tree induced by tip nodes that are mapped to taxa in $\mathcal{L}(i)$ and the most recent common ancestor of those leaves, and any other node that is an ancestor of some but not all of these leaves.

**Figure 1:**
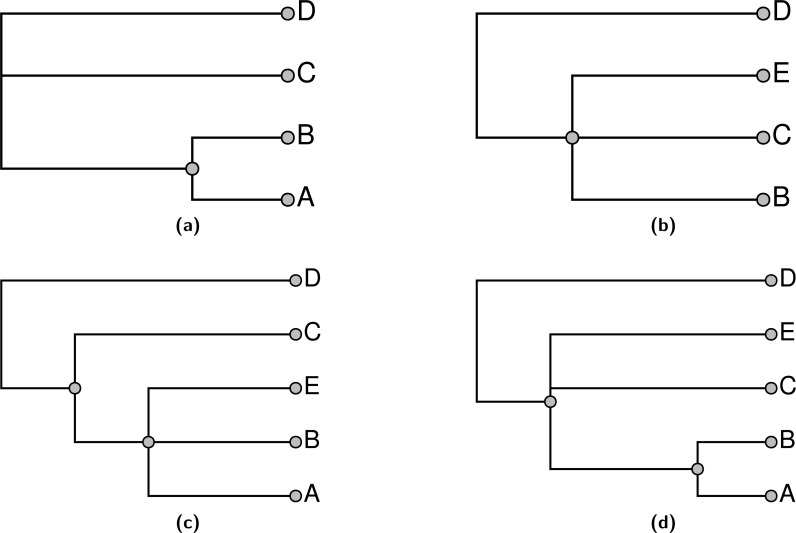
An example demonstrating that our definition of “supported by” does not imply entire composition of a grouping. (A) and (B) show 2 input trees and (C) and (D) depict trees that each display each of the groupings in the input trees and which have no unsupported nodes. The BUILD algorithm (‘Subproblem solution’) would choose tree (D) that floats taxon E closer to the root.

Note that stating that a node in the summary tree is supported by an input does not imply that every descendant of that node must be present in the input nor that every taxon that is not a descendant must be excluded in order to display the node. Consider the problem shown in Fig. 1; Figs. 1A and 1B show two input trees. Because taxa A and E do not occur together in either input, there is some uncertainty about where to place them. By our terminology, either output shown in Figs. 1C or 1D would be characterized as a tree that displays all of the input groupings and which has no unsupported groups. Clearly these criteria are insufficient to specify a unique solution, and users of the output tree need to be aware that it may be possible for some taxa to “float” to multiple positions. In Fig. 1, taxon E floats to different positions in Figs. 1C and 1D, whereas taxon A does not.

One of our aims in supertree construction is to minimize the amount of information in the supertree that does not come from input trees. We permit information that comes from combinations of input trees, but not any single input tree. However, we seek to exclude information that comes from none of the input trees. This motivates the criterion of not having any unsupported edges, since these edges could be removed without decreasing the support from any input tree.

#### Goal 2: Tree ranking

**Figure 2:**
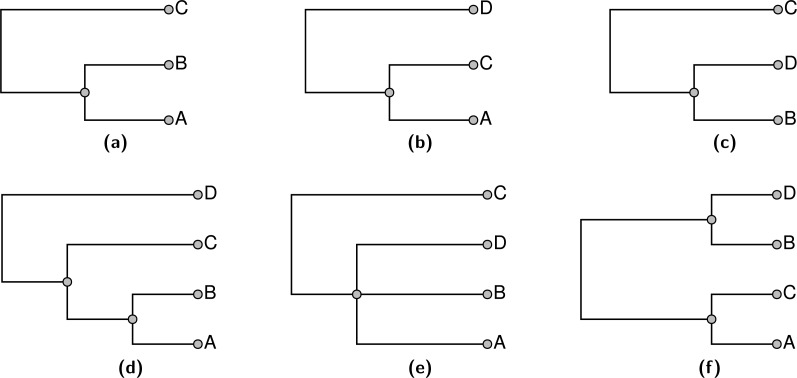
An example of three input trees shown in (A), (B), and (C) which do not conflict in a pairwise manner, but cannot be jointly displayed in one tree. The 3 solution trees are shown in (D–F). (D) for ranking the tree in (C) lowest. (E) shows the solution if the tree in (B) has the lowest rank. (F) shows the solution if the tree in (A) is ranked lowest. Each of the solutions displays two of the three input groupings.

An appealing goal for the summarization would be to find the supertree that displays the largest number of input tree edges. As discussed in Huson, Rupp & Scornavacca (2010; pages 92 and 131) the maximum compatibility problem is known to be *NP*-hard via a reduction from Max-Clique (Karp, 1972). In addition to being computationally daunting, this formulation of the supertree problem does not provide biologists who use the summarization tool with an obvious avenue for fixing perceived problems with the summary tree. For example, a grouping that a biologist expected may not be present in the supertree, but it may not conflict with any of the input groupings which are displayed. This can happen because displaying both node *a* from *T*_1_ and node *b* from tree *T*_2_ in a summary tree may only be possible by displaying a grouping that is present in no input tree. All other factors being equal, if this implied grouping conflicts with input node *c* in tree *T*_3_, then *c* will not be displayed in the summary tree, but a biologist will not necessarily know how to fix this problem. One solution is to use a ranking of groupings. If an expert were quite confident in the *c* grouping, then she could assign that input node a high ranking. A supertree that used ranks could then recover this grouping even if its inclusion did not increase the total number of input nodes that are displayed by the summary tree. Figure 2 shows an example of three input trees for which there is no pairwise incompatibility, but no solution displays all of the input groups. Alternative rankings of inputs can result in one of three summary trees shown in Figs. 2D–2F.

Any approach to supertree construction must deal with the need to adjudicate between conflicting input trees. We choose to deal with conflict by ranking the input trees, and preferring to include edges from higher-ranked trees. The merits of using tree ranking are questionable because the system does not mediate conflicts based on the relative amount of evidence for each alternative. However, it is a reasonable starting point. It has the benefits of making it easy to see why some groups are included or not (transparency), and it allows simpler and cleaner algorithms.

Note that if some edge *c* conflicts with a higher-ranked edge *b*, then *c* may still be included in the supertree. This can occur when the higher ranked edge *b* conflicts with a yet-higher ranked edge *a*, and thus *b* is not included. In that case, it will be possible for *c* to be represented in the summary tree. Thus, the fact that the summary tree displays an input edge does not imply that none of the higher ranked input trees conflict with that edge.

In order to produce a comprehensive supertree, we also require a rooted taxonomy tree in addition to the ranked list of rooted input trees. Unlike other input trees, the taxonomy tree is required to contain all taxa, and thus has the maximal leaf set. We make the taxonomy tree the lowest ranked tree. In our current formulation, the taxonomy tree is also unique in that the taxonomy is the only source of taxonomic names. Each node in the taxonomy tree corresponds to a named group. Taxonomic groups may have the same name, but each node in the taxonomy tree is identified by a unique number (its OTT ID). Taxonomic groups are identified in the summary supertree by finding a branch (or “node”) that has exactly the same include|exclude relationship. The taxonomy supertree can meaningfully possess degree-two nodes. Although these nodes can be removed without affecting the relationships of the leaves, they do represent nested taxonomic groups that contain exactly one subgroup. The taxonomy is also used to determine which tips are terminal taxa.

#### Goal 3: contain no unnecessary polytomies

The supertree should be as resolved as possible—in other words, it should have no unnecessary polytomies. Thus, for each input edge that is *not* included, we can point to a reason for non-inclusion by showing that the input edge conflicts with some edge of the summary tree. Note, that the requirement to not display unsupported groups leads to some “necessary” polytomies. For example any resolution of the polytomy shown in Fig. 2E would continue to display the same two input groups. However, the additional grouping would be unsupported, because the unresolved tree already displays both input groups. Thus, the unresolved tree would be preferred by our criterion. However, collapsing either internal edge of the tree shown in Fig. 2D would result in a tree which displays only one input grouping. This tree would contain an unnecessary polytomy, because the polytomy would permit refinement to the depicted tree which displays more input groupings.

#### Goal 4: display as many input nodes as feasible

We also seek to construct a supertree that represents as many input tree nodes as possible. Since non-included input tree nodes must conflict with the supertree (or they would have been added), this criterion is the same as minimizing the number of input nodes that conflict with the supertree.

#### Non-goal: phylogeny estimation

Many researchers construct supertrees for the purpose of inferring the true tree from the input trees (Bininda-Emonds et al., 2007; Davis & Page, 2014). Such approaches treat input trees as data or as surrogates for data matrices (Gatesy & Springer, 2004), and the supertree is seen as more accurate than the individual input trees because more input trees (and presumably more data) stand behind it. For such methods, conflict resolution is a primary aim of the method.

Our supertree method operates in a different paradigm. Phylogeny estimation is an explicit non-goal of our supertree algorithm. We do not claim that the supertree is more accurate than the input trees it summarizes, and so conflict resolution is not a primary aim of the method. Most of our input trees have non-overlapping taxon sets, and so conflict resolution is not the primary problem. Instead, we seek to merge and aggregate phylogenetic information on the largest possible scale. Therefore, the primary aim of our supertree method is to *summarize and represent* the input trees, even when they conflict with each other. The annotations file constructed by our pipeline is essential for this task because it includes information about conflict with our supertree as well as support for it.

Our method must resolve conflicts in order to construct a single supertree. However, the rank information used to resolve conflicts is an input to the method, not an output from the method. We thus perform curation-based conflict resolution, not inference-based conflict resolution.

#### Summary of goals

These optimality criteria help to define what it means for the supertree to represent the input trees, as well as justifying and explaining why various features of the supertree exist. The pipeline described below produces a supertree that satisfies the first three optimality criteria and is a greedy approximation of a solution to the fourth goal. It is not guaranteed to display as many input nodes as possible. Even if the summary tree does accomplish goal 4, it is not necessarily a *unique* optimum. The pipeline takes a greedy approach to producing a summary tree by attempting to add groupings from the trees in order of the trees ranking. This can be viewed as a greedy solution to the problem of finding the tree with the maximum sum of displayed groups’ weighted scores criterion (MSDGWS, described in the Appendix S1) where the weights from the trees are so extreme that displaying one group from a highly ranked tree is preferred to displaying all of the groupings from lower ranked trees. These large weights help to achieve our goal of allowing curators to fix problems in the tree. While it is possible to view our pipeline as an optimization algorithm by assigning weights to the input trees in this way, we are not trying to weight the evidence in the input trees, and we do not actually ever calculate these weights in our pipeline. Note that in this extreme form of weighting, we do not have to calculate the score of a tree, we simply traverse the groupings by order of rank and add a group if it is compatible with the previously added groups.

#### Comparison to other supertree methods

Matrix Representation Parsimony (MRP) is one of the most widely used supertree approaches (Baum, 1992; Bininda-Emonds et al., 2007; Davis & Page, 2014). MRP attempts to encode branches of input trees as characters in a data matrix, and then find an optimal tree according to the maximum parsimony criterion. Such an approach does not provide a clear and transparent explanation for including particular edges in the supertree. MRP can also infer edges that exist in none of the input trees, which violates our Goal #1 (Gatesy & Springer, 2004).

Another commonly used method is MinCut (Semple & Steel, 2000) or Modified MinCut (Page, 2002). Like our approach, MinCut is based on the BUILD algorithm (Aho et al., 1981). However, MinCut deals with conflicts by modifying the BUILD algorithm to resolve incompatibilities by discarding edges that are present in the smallest number of input trees. This approach thus violates our Goal #2 of resolving conflict via ranks that can be altered by a curator to influence the output tree.

## Description of the Supertree Method

### Preprocessing steps

**Figure 3:**
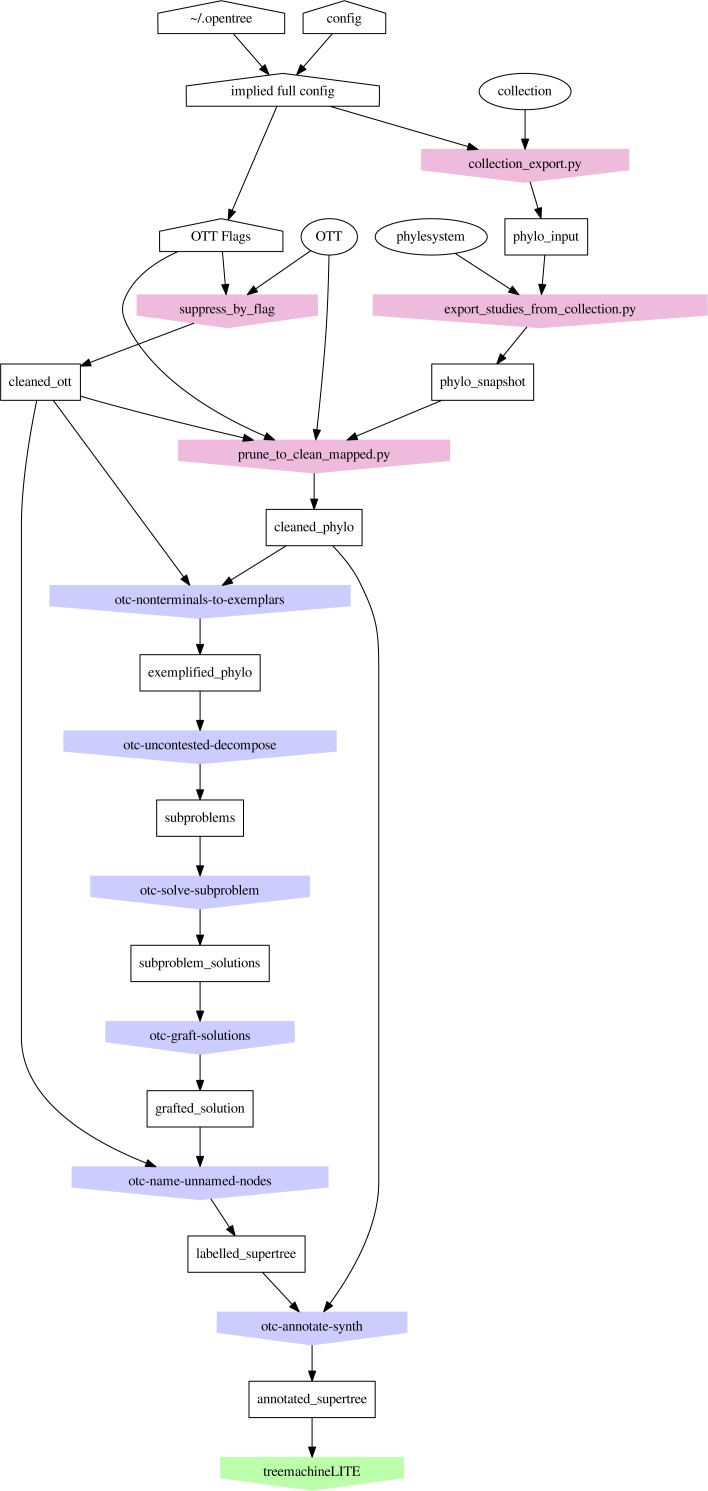
Organization of the *propinquity* pipeline. Each colored pentagon labels a program (blue for otcetera-based tools and red for python scripts in the propinquity or peyotl repository) that performs the important operations in each step; the number before the tool name refers to the section in this paper that describes the operation. The output of each step corresponds to a subdirectory of the propinquity system which will hold the output artifacts for the step. Ovals are resources that are required (OTT and Open Tree’s phylesystem repository). White pentagons are user-controlled inputs.

Propinquity was designed to function as a part of the Open Tree of Life software architecture, so the first few steps of the pipeline involve transforming artifacts from that project into a set of rooted trees and a phylogenetic taxonomy. The phylesystem API (McTavish et al., 2015) of Open Tree allows users to curate published estimates of trees and create ranked collections of these trees. Early steps in the propinquity pipeline manipulate the phylogenetic input trees to improve their usability and reliability. The first steps of the pipeline (see Fig. 3) collect a list of trees to include (in the phylo_input subdirectory) and store copies of these files (in the phylo_snapshot subdirectory) to make it easier to replicate the operation (because the collection of trees and the tree files change due to curation).

#### Pruning questionable taxa from the taxonomy

OTT is a hierarchy of taxonomic names that implies a phylogenetic taxonomy. An OTT ID has a position in the hierarchy, a taxonomic name, and set of references to the same name in different taxonomies. In addition, the ID may also be associated with a set of flags that can indicate that the taxon may be questionable. These flags can either encode information taken from an input taxonomy (for example, taxa the NCBI refers to as “unplaced” are assigned an “unplaced” flag) or can arise because of some form of conflict during taxonomy construction (for example, if two taxonomies disagree on the name for a taxon, then the taxon can be merged and the name will be retained without any descendants; this name will have an OTT ID, but will be flagged as “barren”). Propinquity prunes the OTT down to a more reliable taxonomy by pruning off parts of the tree that are flagged with suspicious flags. The set of flags that lead to a subtree of the taxonomy being pruned is under the control of the user (the set of flags used by the Open Tree of Life project can be found in the config.opentree.synth file in the propinquity repository). For the purpose of the rest of the pipeline, an OTT ID that has been pruned from the taxonomy will be treated in the same way as invalid OTT ID. The output of this step is stored in propinquity’s cleaned_ott subdirectory; this operation only needs to be performed when the OTT or the pruning flags change.

#### Pruning problematically mapped tips from input phylogenetic trees

Frequently, phylogenetic estimates are rooted using the outgroup criterion, which is an assumption about the monophyly of the ingroup taxa. Because the rooting of the branches in the outgroup portion of the tree is often uncertain, data curators can identify the ingroup node of the tree; propinquity uses this annotation to prune off the outgroup taxa.

Frequently, not all tips in a phylogenetic input will have been mapped to a taxon in the current version OTT. Unmapped leaves are pruned from each phylogenetic input. In some cases, the OTT has changed and a taxon has been unambiguously mapped to another taxon. This can occur when multiple species in one version of the taxonomy are “lumped” into a single taxon in a subsequent version. OTT maintains a set of “forwarding” statements about IDs that have been removed but can be mapped to an existing taxon; propinquity uses these statements to update the OTU mapping of input trees.

Finally some leaves are mapped to taxa that occur more than once in the tree, or taxa that have ancestors represented as tips of the tree. In these cases, leaves are pruned to assure that tips are mapped to unique taxa that are not nested. In the case of nested taxa, the tip mapped to the higher level taxon is pruned, and one of the lower level tips is retained. In the case of duplicate occurrences of an OTT taxon, propinquity checks to see if a data curator has selected one of the taxa to be the exemplar for the taxon. If this selection has not been made, then the node with the lexicographically lowest ID is chosen to exemplify the taxon. This choice is arbitrary, but repeatable. The pruned phylogenetic inputs are stored in a cleaned_phylo subdirectory of propinquity.

#### Exemplifying tips mapped to higher taxa

Many input trees have tips that are not terminal taxa, but higher-order taxonomic groups. It is not clear how to interpret a tip in a phylogenetic estimate that is labeled with the name of a higher taxon. Several scenarios can lead to these cases: the data for the tip could have been created by merging a chimeric set of character scores from constituent taxa; the species sampled may not have been identified to the lowest taxonomic rank; or the researcher may simply have used a higher taxonomic name because he/she assumed that the taxon is monophyletic and the higher level name would be more recognizable. Rather than allowing the ambiguity about interpretation of the higher-taxon mapped tips to propagate throughout the entire pipeline, we transform the input trees by replacing higher taxa at tips with a set of terminal-taxon exemplars for each taxon. One approach would be to simply determine all descendant terminal taxa and attach them as children of the problematic tip. However, this would create a clade rather than a tip; subsequent steps in the supertree would interpret the clade as a claim of monophyly for the taxon. The input tree may not have tested monophyly of the clade, so this interpretation is unwarranted. We avoid it by attaching exemplar taxa as child nodes of the higher taxonomic tip but then collapsing the edge between the former tip node and its parent. Thus, if *A* is a non-terminal taxon containing terminal descendants *a*_1_ and *a*_2_ and *B* is a non-terminal taxon containing terminal descendants *b*_1_ and *b*_2_ we would replace the subtree ((*A*, *B*), *c*) with ((*a*_1_, *a*_2_, *b*_1_, *b*_2_), *c*) instead of the subtree (((*a*_1_, *a*_2_)*A*, (*b*_1_, *b*_2_)*B*), *c*).

**Figure 4:**
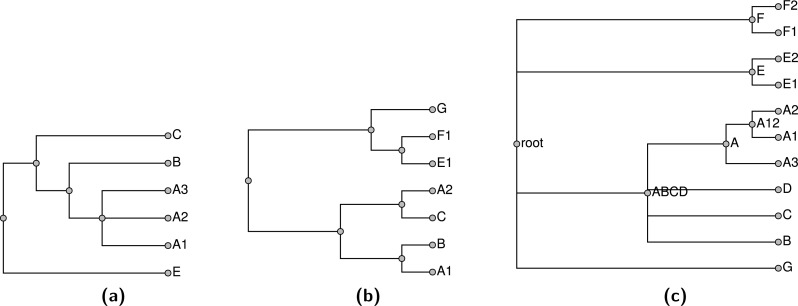
Input trees (A–B) and taxonomy tree (C).

**Figure 5:**
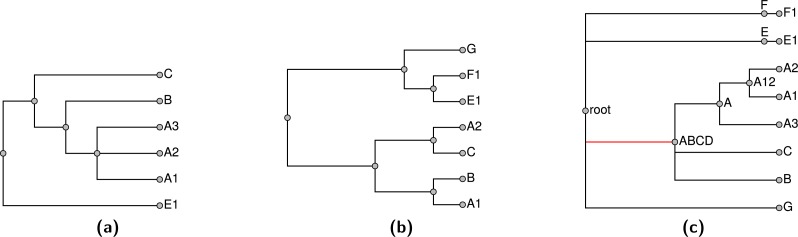
Exemplified input trees (A–B) and pruned taxonomy tree (C) from Fig. 4. Taxon E in the first input tree is exemplified by E1 in (A). Pruned taxa are E2, F2, and D. The taxa E and F are retained as monotypic taxa in the pruned taxonomy. The red edge in the pruned taxonomy tree is an uncontested higher taxon in the exemplified taxonomy (as explained in section ‘Subproblem decomposition’ ).

If a taxon is only present in the taxonomy (not in any of the input trees), then it can be pruned from the taxonomy for the construction of the supertree and then grafted back on to the summary tree later. Performing this pruning reduces the size of the supertree problem, reducing the running time of the pipeline. Similarly, when we expand a higher taxon in the exemplification step, we can omit members of the taxon if they do not occur in any of the phylogenetic inputs. If there are no members of the higher taxon sampled in any other input tree, then we arbitrarily choose one terminal taxon to represent the higher taxon. During the exemplification step, a tool from otcetera (otc-nonterminals-to-exemplars) reads the taxonomy and all of the “cleaned” phylogenetic estimates from the previous step. Reading all of the inputs is necessary to assure that each higher taxon is replaced with the same set of exemplars regardless of which tree the higher taxon occurs in, and that the exemplars for a higher taxon is the union on the set of descendant terminal taxa that have been sampled in a phylogenetic input. For example, the trees in Fig. 4 would exemplified as shown in Fig. 5.

We prune the taxonomy by removing tips that are not present in any input tree to produce the pruned taxonomy 𝕋_*p*_. The tips pruned in this step will be grafted back onto the skeleton of the summary tree in a subsequent step. A terminal taxon that is represented only in the taxonomy can be pruned and then regrafted onto the solution without affecting which nodes are displayed by the final summary tree. Thus, this procedure does not impede our ability to find a good summary tree. Removing these tips produces a smaller input to the rest of the pipeline, which reduces running times. After producing the set of “exemplified” phylogenetic inputs, this tool exports a pruned down version of the taxonomy that only contains tips that are present in at least one phylogenetic input.

### Summary tree construction

After the preprocessing steps, the inputs have been converted to a set of rooted phylogenetic estimates in which each leaf is mapped to a terminal taxon in the exemplified taxonomic tree. The goal of the remainder of the pipeline is to construct a tree that maximizes the sum of displayed groups’ weighted scores (MSDGWS) criterion. This is accomplished in four steps: (1) dividing the full problem into subproblems based on uncontested taxa; (2) constructing a summary solution for each subproblem by greedily creating a maximally-sized list of groupings that can all be displayed simultaneously; (3) grafting the subproblem solutions into a single supertree; and (4) grafting (or “unpruning”) the taxonomy-only taxa onto the solution to produce a complete summary tree.

#### Subproblem decomposition

For the sake of efficiency, propinquity uses a divide-and-conquer approach to construct the supertree. Subproblems are identified by searching through the taxonomy tree to find any taxa that are not contested by any single input tree. Here we say that input tree *T*_*i*_ contests taxon *x* in the pruned taxonomy, if *x* is not monophyletic in any resolution of tree *T*_*i*_. Thus, polytomies in an input tree are treated as soft polytomies, and a taxon is not contested merely because it is not displayed by an input tree.

**Figure 6:**
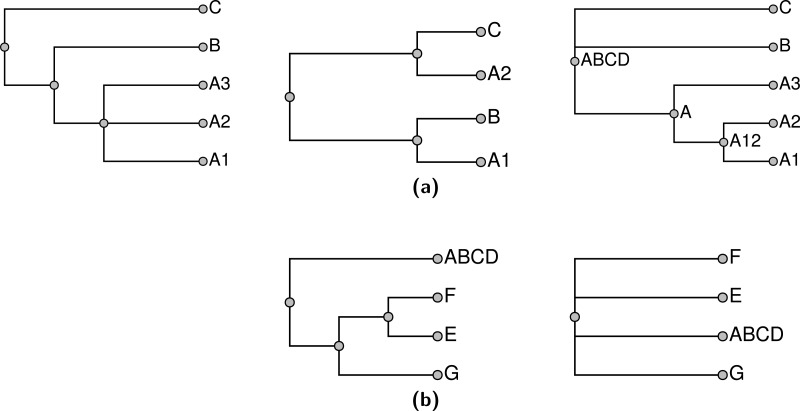
Subproblems (A) *ABCD* and (B) *root* generated from the exemplified trees shown in Fig. 5. A trivial statement from the first tree that a taxon labelled *ABCD* is sister to *E* has been omitted, because trees with only two leaves do not contain phylogenetic information.

This operation is performed by the otc-uncontested-decompose tool in otcetera; see Appendix S2 for a description of the algorithm. The output is a series of subproblems, each of which corresponds to a slice of the taxonomy and corresponding slices through each relevant input tree. Each uncontested non-terminal and non-root taxon will show up in two subproblems: it will be the root of its own subproblem and it will be tip in the subproblem that covers the next slice deeper in the tree. The red edge in Fig. 5C highlights the taxa that are not contested by the input shown in Fig. 5; Fig. 6 shows the subproblems that would be emitted as a result of this set of inputs. The supertree operation of Hinchliff et al. (2015) also used this otcetera-based decomposition step.

**Figure 7:**
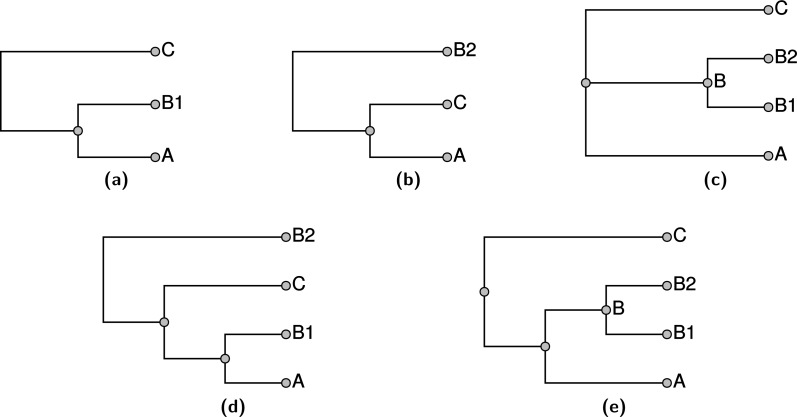
An example with three input trees: the highest ranked phylogenetic input (A), the second ranked phylogenetic (B), and the taxonomy in (C). The summary tree in (D) has the highest possible score, but the summary shown in (E) would be returned from the pipeline that uses uncontested taxon decomposition.

Note that decomposition into uncontested groups does not necessarily allow us to find the tree that maximizes the MSDGWS score. For example, see Fig. 7; that example is a variant of the situation shown in Fig. 2. In this case the groupings from each of the phylogenetic estimates, shown in Figs. 7A and 7B, could be displayed. That solution is shown in Fig. 7D, it displays two of the three input splits, but is optimal because no solution displays all three input groupings and the depicted solution displays the two highest ranked groupings. However, neither of the trees shown in Figs. 7A or 7B contest the taxon *B* shown in the taxonomy panel Fig. 7C. Thus, when using our decomposition, the branches leading to taxa *B*1 and *B*2 in the input phylogenetic trees would be sliced during the decomposition, and relabeled to refer to taxon *B*. This taxonomically-informed interpretation of the inputs views the two phylogenetic inputs as in conflict; so the solution returned by propinquity would defer to the higher ranked tree. The tree shown in Fig. 7E would be returned. This example arises from the fact that the trees in Figs. 7A and 7B jointly contest taxon *B*, but neither contests taxon *B* when the trees are considered in isolation.

Despite the fact that the use of otc-uncontested-decompose can worsen the final score of the summary tree, we use this approach in propinquity because it makes the construction of the tree faster and it is easy for users to correct issues caused by incorrect taxa being constrained to be monophyletic. By adding a tree (even a low-ranked tree) that contests a taxon to the corpus of input trees, then the next synthetic tree will no longer consider the taxon to be uncontested. Thus the procedure encourages curation of more phylogenetic inputs as a means of improving the summary tree.

**Figure 8:**
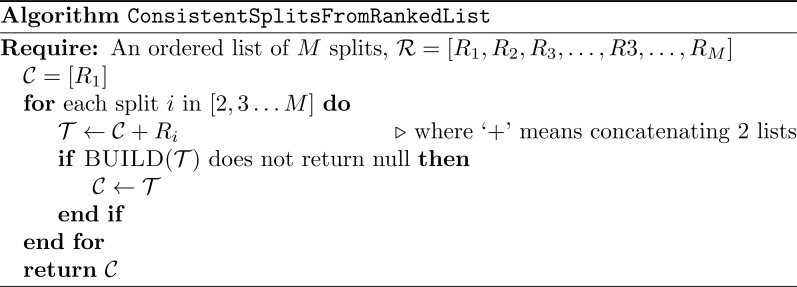
Algorithm ConsistentSplitsFromRankedList.

#### Subproblem solution

When solving sub-problems, we sequentially incorporate splits from trees in order of ranking, retaining splits that are compatible with the current set of splits (Fig. 8). The order of splits from the same tree is not specified by this approach, and we incorporate splits using one of the possible post-order traversals of the tree. We make use of the BUILD algorithm (Aho et al., 1981) to assess compatibility. This strategy avoids unnecessary polytomies, since splits of later input trees are only rejected from the summary supertree if they conflict with higher-priority splits. Finally, we use the BUILD algorithm to construct a supertree displaying all of the splits in the set of compatible splits. Using the BUILD algorithm to construct the subproblem summary tree satisfies criterion 3, because trees from the BUILD algorithm do not contain unsupported branches.

**Figure 9:**
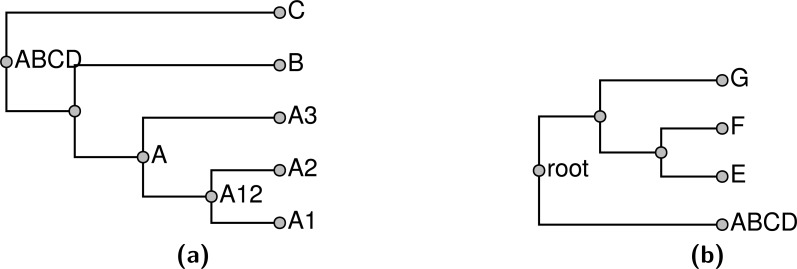
Solutions to (A) subproblem *ABCD* and (B) subproblem *root* depicted in Fig. 6.

The BUILD algorithm as originally stated by Aho et al. (1981) applies to a collection of rooted triplets. Instead of decomposing each input split into a collection of rooted triplets, we instead modify the BUILD algorithm to apply directly to larger rooted splits. The modified BUILD algorithm constructs a tree compatible with a collection of rooted splits, and returns failure if such a tree does not exist. This modified algorithm recovers the original BUILD algorithm if only rooted triplets are supplied as input. When larger splits are supplied as input, the results are the same as if each was was decomposed into all implied triplets. The modified build algorithm has order *O*(*N*^2^ + *N*^2^*E* + *NL*) where *N* is the number of splits passed in, *E* is the average size of the exclude group, and *L* is the total number of leaves. This simplifies to *O*(*N*^2^) if all splits are triplets. In this approach splits are either entirely retained or entirely discarded—consistent rooted triplets from conflicting splits are not retained. However, when unpruning taxonomy-only taxa (see below), we make an attempt to break ties in a way that preserves some partial information from conflicting splits by attaching taxa from conflicting splits at their common ancestor. Figure 9 shows the solutions that would be obtained by applying our modified version of the BUILD algorithm to the subproblems shown in Fig. 6.

#### Solution grafting

**Figure 10:**
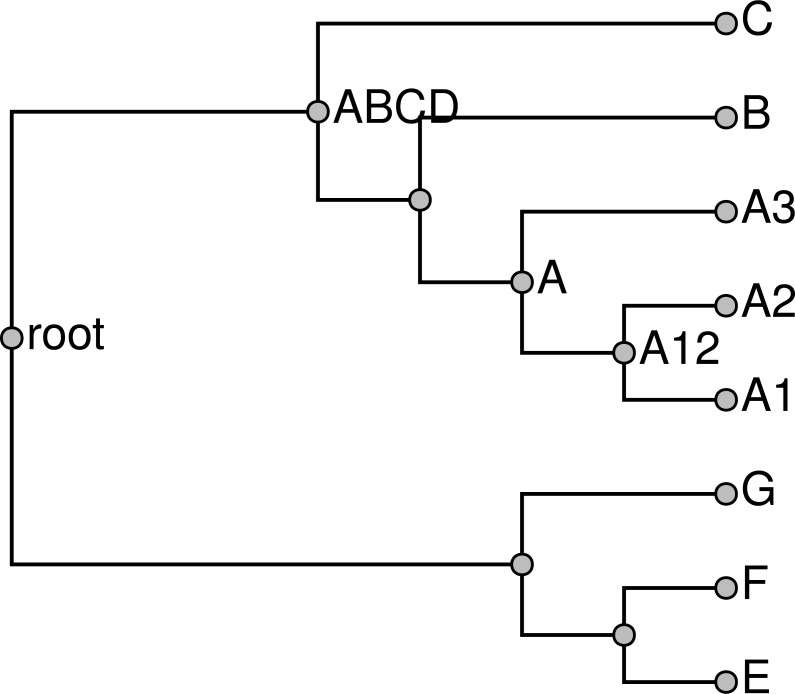
Grafted solution produced from the subproblems from Fig. 9 and which is the backbone onto which taxa that are not included in any phylogeny will be placed.

To produce a tree that spans all of the taxa sampled in the exemplified set of input trees, we graft the subproblem solutions into a single tree (stored as the grafted_solution/grafted_solution.tre by propinquity); see Fig. 10. Recall that each non-root uncontested taxon used for decomposition occurs as a leaf taxon in one subproblem and as a root taxon in one other subproblem. Thus, the grafting operation simply consists of reading all of the subproblem solutions into memory and then merging the nodes that are labeled with the same OTT ID.

**Figure 11:**
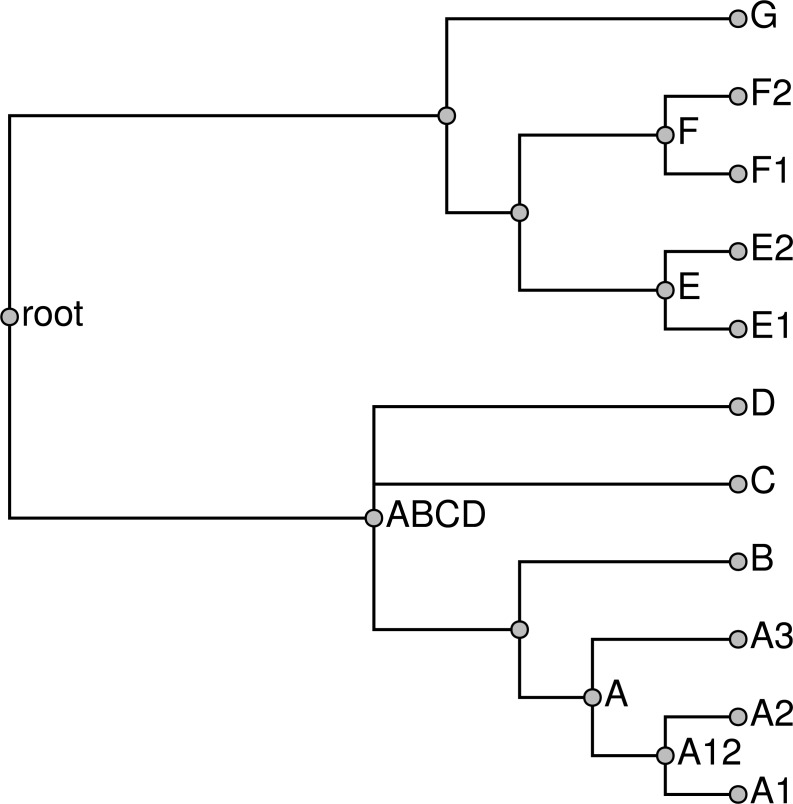
Unpruned tree with internal taxa. Taxa unsampled in phylogenetic statements have been added to the grafted tree shown in Fig. 10.

**Figure 12:**
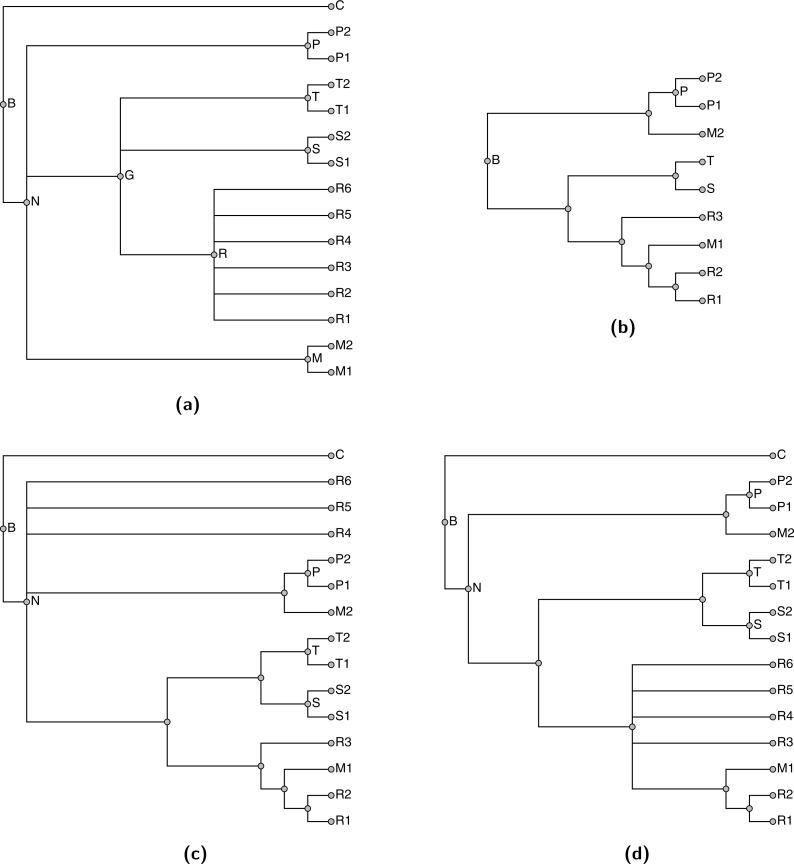
Two approaches to unpruning. Taxa G and R in the taxonomy (A) are broken because they conflict with the grafted solution (B). Removing these broken taxa from the taxonomy before unpruning leads to taxa R4, R5, and R6 being attached directly at taxon N, as in tree (C). In tree (D), the children of the broken taxon R are instead attached at the MRCA of R1, R2, and R3. Our method follows the second approach.

#### Unpruning unsampled taxa

As described above, taxa that do not have any descendants in a sampled phylogenetic input are pruned from the taxonomy for the sake of efficiency. These taxa are reattached by an “unpruning step.” For those taxa that are compatible with the grafted tree, this step simply amounts to adding any unsampled taxonomic children to the node that represents the taxon in the grafted solution tree. For example, the tree in Fig. 10 is unpruned to produce the tree in Fig. 11.

However, a taxon may be incompatible with the grafted solution; we refer to such taxa as “broken taxa.” If a broken taxon contains some unsampled children, it is not clear where these unsampled children should be attached to the grafted solution. One approach would be to mimic the application of Algorithm ConsistentSplitsFromRankedList (Fig. 8) to the full (unpruned) taxonomic tree. This would be equivalent to collapsing each edge in the taxonomy that attaches a broken taxon to its parent. The unsampled children of broken taxa would attach at their least inclusive ancestral taxon which is unbroken. In cases of several adjacent taxa are broken, this can lead to polytomies of very high degree deep in the tree. This can make the summary tree difficult to navigate. Thus, we have adopted an alternative solution. The otc-unprune-solution-and-name-unnamed-nodes tool from otcetera attaches the unsampled children of a broken taxa to the grafted solution as children of the MRCA of the sampled children.

Figure 12 illustrates the two approaches to unpruning. Taxa G, M, and R (Fig. 12A) are broken because they conflict with the grafted solution (Fig. 12B); among these, only taxon R has children that were unsampled in the grafted solution. Ignoring all broken taxa when unpruning would cause the unsampled children (R4, R5, and R6) to attached directly at taxon N (as in the tree shown in Fig. 12C), because that is the least inclusive unbroken ancestor of R. The tree illustrated in Fig. 12D shows the tree that would be produced by propinquity; the children of the broken taxon R and instead attached at the MRCA of sampled children (R1, R2, and R3). Their attachment point does not correspond to any taxon in the taxonomic tree.

#### Naming unnamed nodes

In order to annotate each node in the summary supertree, it is first necessary that each node have a unique identifier. Nodes whose include group correspond exactly to the include group of a node in the taxonomy are given the same identifier as the corresponding taxonomy node. These identifiers are of the form *ottX* where *X* is an integer OTT ID. We generate a label of the form *mrcaottX*_1_*ottX*_2_ for an non-taxonomic node *n* where *X*_1_ and *X*_2_ are the OTT IDs for two leaves, *n* is the MRCA of these leaves, and *X*_1_ is the numerically smallest OTT ID that is a descendant of *n*, and *X*_2_ is the next the smallest ID that can be chosen to designate *n* as the MRCA. Because new taxa added to OTT will be given higher OTT IDs, the use of the lowest numbered OTT IDs as designators increases the chance that a node label can be encountered in a subsequent version of the tree (though the taxonomic content may change). The deterministic choice of designators also makes the labeling insensitive to branch rotation of the grafted solution tree.

#### Annotation

To reveal the connections between the groupings found in the a summary supertree and the input trees, propinquity uses a few Python scripts and the otc-annotate-synth tool from otcetera to create an annotations file describing the pipeline used and the connections between phylogenetic information in the inputs and the summary. The JSON file produced by otc-annotate-synth encodes a “nodes” property that holds a mapping between a node name for the summary tree (using the naming convention described in the previous section) and a node provenance object that categorizes the relationship between the node and the inputs. The node provenance object for node *x* uses several properties to categorize the relationship between the node and the inputs; each property in the node provenance object maps to a structure storing the tree ID and node IDs for the input tree nodes.

Conceptually, this annotation operation is equivalent to considering every node *j* in each input tree *i* and the summary tree node *x*. Because the vast majority of nodes in the input studies will be compatible but not directly relevant to node *x* we do not list all of the compatible groupings. If node *x* is not included in the induced tree 𝕊(*i*), then none of the nodes of tree *i* will be referred to in the annotations for node *x*. Even if *x* is included in 𝕊(*i*), many of the nodes of *T*_*i*_ will be compatible with *x* while being relevant to other parts of the summary tree. The only input nodes listed for node *x* are with rooted taxon bipartitions which conflict with, are displayed by, or are resolved by the the rooted taxonomic bipartition associated with node *x*. All input nodes that cannot be displayed by any supertree that contains *x* are stored in a “conflicts_with” property of the node provenance object. If node *j* of *T*_*i*_ is displayed by the summary tree and *x* is part of the path of 𝕊(*i*) that displays the split between descendants of *j* and other taxa, then a reference to the node *j* will be in the node provenance object. The exact categorization of this annotation will depend on the configuration of node *x* on the induced tree 𝕊(*i*):

• if *x* is on a terminal path in the induced tree then node *j* will be listed in the “terminal” property;
• if *x* is along an internal path that contains some nodes with out-degree equal to 1, then node *j* will be listed in the “partial_path_of” property; and
• if *x* is along an internal path without any node of out-degree 1, then node *j* will be listed in the “supported_by” property, because node *j* supports the existence of grouping *x* in the sense that collapsing the edge that separates *x* from its parent would cause the summary tree to no longer display node *j*.

**Figure 13:**
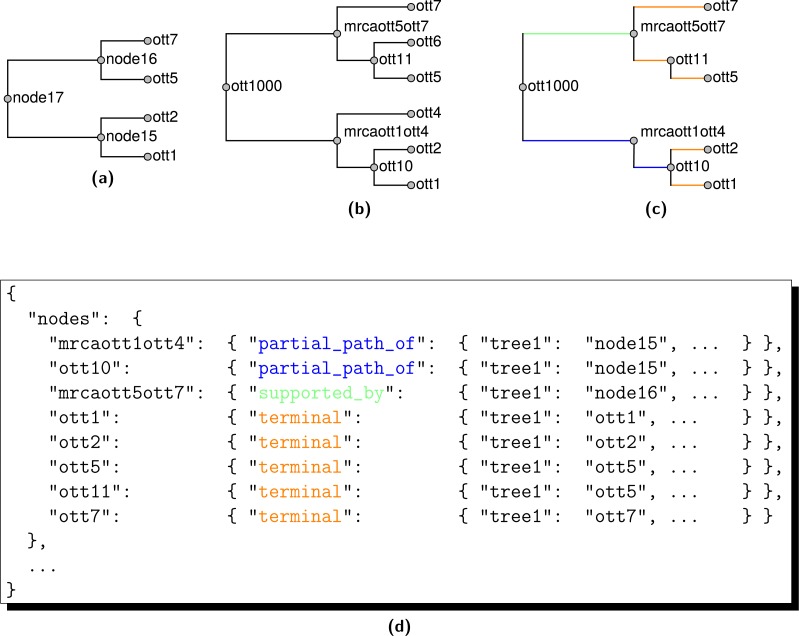
The relationship of edges in summary tree 𝕊 in (B) to edges in the input tree *T*_1_ named “tree1” in (A). Only edges of 𝕊 that are present in the induced tree 𝕊(1) in (C) are represented by JSON annotations in (D). Taxon names are here suppressed in favor of OTT IDs, and edges are referenced via their tipward nodes. Edges in 𝕊(1) that correspond to terminal edges of *T*_1_ are orange; edges of 𝕊(1) that are supported by edges of *T*_1_ are blue; where multiple edges of 𝕊(1) correspond to the same edge of *T*_1_ they are green. There is no conflict in this example. Also, if this were output from propinquity, then each internal node of 𝕊 would be supported by other inputs trees that are not shown here.

These three relationships are illustrated in Fig. 13. Finally, if *T*_*i*_ does not display *x* from 𝕊(*i*), but there exists an unresolved node *j* in *T*_*i*_ which could be resolved such that the tree would then display *x*, then a reference to node *j* will be listed in the “resolves” property of node *x*.

The otcetera annotation tool can also detect cases in which including information from node *j* in *T*_*i*_ could further resolve a polytomy *x* in the summary tree; such a case would be annotated using the “resolved_by” property of *x*. However, because of our goal of excluding unnecessary polytomies, none of the nodes in propinquity’s summary tree will use this annotation when they are annotated with the set of input trees.

#### Self-documentation

An optional step in the propinquity pipeline (triggered by the executing the “make html” target) can compose an “index.html” file for each directory created during the pipeline to explain the artifacts held in that directory and report summary statistics about the summarization run.

## Results

We seek to assess the performance of our new supertree method by comparing it to the supertree method of Hinchliff et al. (2015). The method of Hinchliff et al. (2015) was used to construct the Open Tree of Life v4 (OTLv4). Therefore, in order to facilitate comparison, we applied our method to the same input trees and taxonomy used by OTLv4. We refer to the resulting supertree as OTLv4′ since it was constructed by applying the propinquity pipeline to the same inputs as OTLv4.

### Inputs

The flag-cleaned version of OTT used in the construction of both supertrees contained 2,424,255 leaves. The OTLv4 supertree was constructed from 482 phylogenetic inputs, containing a total of 45,385 leaves, of which 41,029 were unique. After flag-cleaning and exemplification by propinquity, these trees contained 40,323 unique tips. We used the same cleaning flags to trim the taxonomy and input trees when constructing OTLv4′, so OTLv4 and OTLv4′ contain the same number of leaves.

### Subproblems

In the OTLv4′ summary tree, the decomposition procedure produced 5,406 subproblems, but only 1,422 of these were non-trivial to solve. If a subproblem contains only two tips it is trivial; 2,362 subproblems were trivial in this way. Similarly, if a subproblem contains only 2 trees it is trivial to solve because the solution will simply be all of the groupings from the first tree combined with all of the groupings from the second tree that are compatible with the first tree; 3,052 subproblems were trivial in this way. The subproblem with the largest number of tips contained 946 tips. The largest subproblem, in terms of the number of input trees (including the taxonomic tree) that were relevant, had 16 trees. Without decomposition, the supertree problem would have had 482 input trees and 41,226 leaves.

### Representing input splits

We performed an annotation of both the OTLv4 tree and the OTLv4′ tree as described in section ‘Annotation’ to assess the ability of our new supertree method to represent splits from input phylogenies. Table 1 classifies the input phylogeny splits according to how they relate to a summmary tree, so that each input edge falls in one of supported_by, partial_path_of, terminal, conflicts_with, or resolved_by. For example, the numbers in the conflicts_with column indicate the number of input splits *j* with at least one summary edge *x* such that the relation “*x* conflicts_with *j*” holds. The total number of non-terminal input phylogeny splits considered was 40,996.

**Table 1:** Representation of input splits in the OTLv4 tree and the OTLv4′ tree.

	supported_by	partial_path_of	terminal	conflicts_with	resolved_by	resolves
OTLv4	34,595	923	45,385	2,760	2,718	473
OTLv4 only	745	54	0	2,055	2,718	0
OTLv4′	38,521	1,118	45,385	1,357	0	515
OTLv4′ only	4,671	249	0	652	0	42

The number of displayed input splits (supported_by + partial_path_of) increased from 35,518 (for OTLv4) to 39,639 (for OTLv4′); an 11% increase. When examining which splits are displayed, we find that the OTLv4′ tree displays 4,920 input splits that are not displayed by the OTLv4 tree, whereas the OTLv4 tree displays only 799 input splits that are not displayed by the OTLv4′ tree. The number of input splits that conflict with the summary (conflicts_with) dropped from 2,760 to 1,357, a decrease of 1,403, or 51%. In accordance with the goal of not containing unnecessary polytomies, the number of input splits that do not conflict with the summary tree, but are not incorporated (resolved_by) dropped from 2,718 to 0. We also find that the number of polytomies in input phylogenies that are resolved by the summary tree increases from 473 for OTLv4 to 515 for OTLv4′.

We also performed an annotation of the OTLv4 tree and the OTLv4′ tree to assess the degree to which these trees represent taxonomy splits (Table 2). The OTLv4′ tree conflicts with 281 more taxonomy splits than the OTLv4 tree. Since the taxonomy is the lowest ranked input tree, this increased conflict with the taxonomy is an expected result of incorporating more splits from higher-ranked input phylogenies.

**Table 2:** Representation of taxonomy splits in the OTLv4 tree and the OTLv4′ tree.

	supported_by	partial_path_of	terminal	conflicts_with	resolved_by	resolves
OTLv4	125,384	0	2,424,255	1,998	4	3,676
OTLv4 only	296	0	0	19	4	17
OTLv4′	125,107	0	2,424,255	2,279	0	3,883
OTLv4′ only	19	0	0	300	0	224

### Checking reliability of the method

To test the reliability of our methods, we divided the OTLv4′ summary tree into a collection of input trees. The input trees were obtained by splitting the grafted tree at each taxonomy node, so that each input tree has a taxonomy node at the root, and taxonomy nodes as tips, but no taxonomy nodes internally. Each leaf node that was an internal taxon was then replaced with a leaf taxon. This approach ensures that any taxa that were contested in the original analyses remain contested in this new analysis. We then constructed a summary tree from these input trees using the propinquity pipeline. The resulting summary tree was identical to the OTLv4′ summary tree.

### Sensitivity of the OTLv4′ supertree to ranks

We ran the pipeline on the OTL4 data with the ranks reversed. This drastic alteration of the ranks led to a summary supertree that conflicted with 1,264 branches of the OTLv4′. Compared to this, the OTLv4 supertree tree conflicts with 1,387 branches of the OTLv4′ supertree. Thus, reversing the ranks makes a smaller different in the OTLv4′ supertree than the new synthesis algorithm. We also note that a total of 36,533 edges of the grafted tree could be altered by changing the ranks, and most of these edges are unaffected.

## Conclusions

Here we have described the motivation and methodology used by our new supertree method that is currently used by the Open Tree of Life project to build summary supertrees on the scale of millions of leaves. Our new method represented 11% more input phylogeny splits with 51% less conflict compared to the Open Tree of Life version 4 summary tree, when applied to the same inputs. Unlike the previous method (Hinchliff et al., 2015), our new method is guaranteed to incorporate input splits unless they conflict with the summary tree. The method is implemented in the Open Source software package propinquity. A modified version of the treemachine software which built the summary tree described in the Hinchliff et al. (2015) paper is used by the project to serve the tree produced by propinquity via Open Tree of Life APIs.

Our supertree pipeline makes it possible to summarize and merge information from across the Tree of Life. However, we do not claim that our supertree is more accurate than the input trees that it summarizes. Before using the output of our supertree pipeline as input to an evolutionary analysis, researchers should first assess the accuracy of the output supertree in the clade of interest, possibly adjusting the ranks of the input trees to produce a more useful outcome. We have demonstrated that ranks supplied by curators have substantial effects on the output of the pipeline. This can be considered to be a benefit of our approach, since it allows curators to adjust the output of the pipeline, and it is easy to see why the supertree contains the relationships that it does. However, one downside of our current approach is that every node in an input tree must recieve the same rank. As a result, bootstrap proportions and posterior probabilities are not taken into account.

The migration of summary tree construction from treemachine (used for version 4) to propinquity (for all versions from v5.0 to present) has increased the pace of synthesis tree releases from the Open Tree of Life project. This is partly because the newly available annotations feature has made it possible to identify which input trees are responsible for taxa being included or excluded from the summary tree. Additionally, the new propinquity software pipeline has decreased the computational time required to construct a supertree from several hours to about 8 min (after some preprocessing steps which only have to be performed when the input taxonomy changes). The amount of RAM required during tree construction has also decreased substantially.

##  Supplemental Information

10.7717/peerj.3058/supp-1 Appendix S1

10.7717/peerj.3058/supp-2 Appendix S2

## Additional Information and Declarations

### Data Availability

The following information was supplied regarding data availability: GitHub: https://github.com/OpenTreeOfLife/propinquity

https://github.com/OpenTreeOfLife/otcetera

https://github.com/OpenTreeOfLife/peyotl.
