# Supplementary material for: A supertree pipeline for summarizing phylogenetic and taxonomic information for millions of species
Source: PeerJ. 2017 Mar 1;5:e3058. doi: 10.7717/peerj.3058 (PMC5335690; doi:10.7717/peerj.3058)
Supplement: Appendix S1 [file peerj-05-3058-s001.pdf]

A THE MAXIMUM SUM DISPLAYED GROUPS' WEIGHTED SCORES CRITERION

Let $\mathcal{W}$ is a weighting function that maps any input tree's internal node to a non-negative number. If $I(\mathbb{S}, i, j)$ is an indicator function that is 1 if summary tree $\mathbb{S}$ displays the node $V(i,j)$ and 0 otherwise then: $SDGWS(\mathbb{S}) = \sum_i \sum_j I(\mathbb{S}, i, j)\mathcal{W}(i,j)$ is the "sum of displayed groups' weighted scores" for a tree where i indexes all of the input trees and j indexes each non-root internal node in tree i. Preference for this tree is referred to as the maximum sum displayed groups' weighted scores criterion (MSDGWS criterion). The summary tree constructed by the propinquity pipeline is a greedy heuristic for finding a tree that maximizes this score when the weights for a node are determined by the tree's weight and the difference in weighting is so large that displaying one node from a highly ranked tree is preferred to displaying all of the nodes in the trees with lower rank.
