# Supplementary material for: A supertree pipeline for summarizing phylogenetic and taxonomic information for millions of species
Source: PeerJ. 2017 Mar 1;5:e3058. doi: 10.7717/peerj.3058 (PMC5335690; doi:10.7717/peerj.3058)
Supplement: Appendix S2 [file peerj-05-3058-s002.pdf]

B DESCRIPTION OF THE DECOMPOSITION ALGORITHM OF OTC-UNCONTESTED-DECOMPOSE

The input is the a ranked list of input trees and comprehensive taxonomy.

B.1 Creation of multigraph of the taxonomy with embedded trees

The tool creates a multigraph by starting with a graph isomorphic to the taxonomic tree. The nodes and edges created in this step will be referred to as the "taxonomic graph." Next, we add nodes and edges to that graph in a procedure that we refer to as "embedding" the input trees into the taxonomy. A node is introduced for each node in an input tree, and these nodes are mapped the MRCA nodes in the taxonomic graph. In other words, for any node y in an input tree with a cluster of descendants, $\mathcal{C}$, we find the most tipward node z in the taxonomic graph that is an ancestor to all of the taxa in $\mathcal{C}$; let $m(y) = z$ refer to this mapping, and $m'(z) = y$ refer to the reverse mapping. Each edge e_{ij} in a source tree i connects ancestor to its descendant, $a(e_{ij}) \to d(e_{ij})$. The edges are introduced into the graph. We also introduce new edges to create a from $m(a(e_{ij}))$ through its descendants to $m(d(e_{ij}))$; we denote this path $p(e_{ij})$ and refer to the edges in the path as "embedding edges for tree i". Note, that this is a path through the taxonomic nodes, while the edge e_{ij} connects source tree nodes. The mapping between e_{ij} and $p(e_{ij})$ is stored, and the edges are labelled with the index i so that it is clear which tree created them. Because the taxonomic tree is highly unresolved, it is frequently the case that $m(a(e_{ij}))$ is the same node as $m(d(e_{ij}))$; in these cases the embedding edge is a loop. This situation occurs whenever edge e_{ij} can resolve part of the polytomy represented by a taxon.

We treat the taxonomy as the lowest ranked input tree. The next step will collapse contested edges in the taxonomic graph. To retain all of the information from the taxonomy we embed the taxonomy into the taxonomic graph as if it were another input tree

B.2 Detection of uncontested higher taxa

After every input tree and the taxonomy tree have been embedded into the taxonomic graph, we perform postorder traversal over the taxonomic graph that underlies the multi-graph. For any internal node (each of which corresponds to a non-terminal taxon) we determine whether or not it is contested by examining each input tree. We can determine tree i contests the taxon represented by taxonomic node x by looking at the parents of all of the "exiting" embedding edges for tree i. These are the set of embedding edges that have x as a daughter and have a parent node that is not x (ergo a parent node that is taxonomically higher than x). If there are more than one parent nodes in this set of exiting embedding edges, then tree i contests that taxon. If there is only one parent node, then the all of the constituent taxa belonging to this taxon that are present in tree i have one parent that is more inclusive; this means that the input tree does not contest monophyly of the taxon.

If the taxon is uncontested, then it may be the case that some input trees do not contest the taxon, but contain polytomies that could be resolved to display the taxon. The cases can be identified by finding multiple exiting embedding edges that have the same taxon y as their parent node. In these cases, a pseudo input tree node is created and becomes the parent node for these edges; this new node is then connected to y as if it had been an input edge. This operation is equivalent to resolving an input tree's polytomy in favor of the monophyly

of the uncontested taxon. This is the only way in which the input trees are modified during the decomposition.

B.3 Collapsing contested taxa from the taxonomic graph

If a taxonomic node x fails the "uncontested" test described in the previous section, then the node corresponding to the taxon is removed from the taxonomic graph and the set of edges (and mappings between input edges and embedded paths) is updated as if this taxon had not been present in when the taxonomic graph was created. This consists of detecting changing any edge in an embedding path that is adjacent to x by replacing the reference to x with a reference to its parent $a(x)$. Note that we do not collapse the edge corresponding to this taxon in the part of the graph that represents the embedding of the taxonomy into the taxonomic graph. Thus, the taxonomy will still claim the monophyly of the taxon. This is relevant if the input grouping that contests taxon x is overruled (in the subproblem solution step) by a higher ranked split. In other words, the fact that the a taxon is contested during the decomposition is not a guarantee that the taxon will not be monophyletic in the final supertree.

B.4 Emitting subproblems

Whenever an uncontested taxon is identified, the appropriate slice of each input trees that intersect with the taxon is written to a file. Then the multigraph is simplified by slicing any off the taxon. This slicing is accomplished by examing all of the exiting embedding edges for the taxon. The descendant taxa of each input tree is relabeled with the identifier of the contested taxa and all of that node's descendants are removed. Thus this input node will act as as if it were a leaf mapped to the uncontested taxon. All descendants of the taxonomic graph are also pruned off.
